# Development of a Method to Determine the Effectiveness of Cleaning Agents in Removal of Biofilm Derived Spores in Milking System

**DOI:** 10.3389/fmicb.2016.01498

**Published:** 2016-09-22

**Authors:** Ievgeniia Ostrov, Avraham Harel, Solange Bernstein, Doron Steinberg, Moshe Shemesh

**Affiliations:** ^1^Department of Food Quality and Safety, Institute for Postharvest Technology and Food Sciences, Agricultural Research OrganizationRishon LeZion, Israel; ^2^Biofilm Research Laboratory, The Hebrew University-HadassahJerusalem, Israel; ^3^Israeli Dairy BoardYehud, Israel

**Keywords:** dairy industry, biofilm, *Bacillus subtilis*, biofilm derived spores, spores removal effectiveness, cleaning-in-place

## Abstract

Microbial damages caused by biofilm forming bacteria in the dairy industry are a fundamental threat to safety and quality of dairy products. In order to ensure the optimal level of equipment hygiene in the dairy industry, it is necessary to determine the biofilm removal efficiency of cleaning agents used for cleaning-in-place (CIP) procedures. However, currently there is no standard method available for evaluating and comparing cleaning agents for use in CIP procedures in the dairy industry under realistic conditions. The present study aims to establish a CIP model system to evaluate the effectiveness of cleaning agents in removal of biofilm derived spores from the surfaces of stainless steel which is the predominant substrate in milking equipment on dairy farms. The system is based on *Bacillus subtilis* spores surrounded with exopolymeric substances produced by bacteria during biofilm formation. The spores applied on sampling plates were mounted on T-junctions protruding 1.5–11-times the milk pipe diameter from the main loop to resemble different levels of cleaning difficulty. The cleaning tests were conducted using commercial alkaline detergents and caustic soda at conditions which are relevant to actual farm environment. The spores removal effect was evaluated by comparing the number of viable spores (attached to sampling plates) before and after cleaning. Evaluation of the cleaning and disinfecting effect of cleaning agents toward biofilm derived spores was further performed, which indicates whether spores elimination effect of an agent is due to killing the spores or removing them from the surfaces of dairy equipment. Moreover, it was established that the presence of extracellular matrix is an important factor responsible for high level of cleaning difficulty characteristic for surface attached spores. In overall, the results of this study suggest that the developed model system simulates actual farm conditions for quantitative evaluation of the effectiveness of cleaning and disinfecting agents and their cleaning and disinfecting effect on removal of biofilm derived spores.

## Introduction

Bacterial contamination can adversely affect the quality, functionality, and safety of dairy products. It appears that the major source of the contamination of dairy products is often associated with biofilms on the surfaces of dairy processing equipment (Flint et al., 1997; Sharma and Anand, 2002a). Biofilms are highly structured multicellular communities, which allow bacteria to survive in hostile environments (Hall-Stoodley et al., 2004; Kolter and Greenberg, 2006). Bacterial cells in biofilms are characterized by increased resistance to antimicrobial agents and cleaning chemicals (Sharma and Anand, 2002a; Shaheen et al., 2010; Checinska et al., 2015). Biofilms found in the dairy production lines contain significant milk residues, particularly protein, and minerals such as calcium phosphate. The persistence of accumulated microorganisms in the form of biofilms on dairy equipment causes pre- and post-processing contamination, leading to lowered shelf-life of products and possible transmission of diseases (Faille et al., 2002; Sharma and Anand, 2002b; Shaheen et al., 2010). Biofilms are not only a potential source of contamination, but can also increase corrosion rate of metal pipes and equipment often used in the dairy industry, reduce heat transfer and increase fluid frictional resistance (Kumar and Anand, 1998). Thus, it becomes increasingly clear that bacterial biofilms are a major concern to modern dairy industry; especially with current trends toward longer production runs, the use of complex equipment, the automation of plants and increasingly stringent microbiological requirements.

Members of the *Bacillus* genus are of the most common bacteria found in dairy farms and processing plants (Sharma and Anand, 2002a; Simoes et al., 2010). Biofilms of *Bacillus* species may contain both vegetative bacteria and spores. Spore formation occurs preferentially when the biofilm is in direct contact with the oxygen in air and a water-saturated atmosphere (Ryu and Beuchat, 2005; Wijman et al., 2007). This corresponds very well to the situation in a milk line. It was reported that biofilm of *Bacillus* species could consist of up to 90% spores (Wijman et al., 2007; Faille et al., 2014). Since spores are much more resistant to heat and chemicals, they are much more difficult to eliminate than vegetative bacteria (Ryu and Beuchat, 2005). Moreover, the biofilm matrix offers additional protection for imbedded endospores, allowing survival, and colonization of the surrounding environment when conditions are favorable (Branda et al., 2001).

The most common *Bacillus* species found in dairy associated environment are *B. licheniformis*, *B. cereus*, *B*. *subtilis*, *B. mycoides* and *B. megaterium* (Andersson et al., 1995; Bartoszewicz et al., 2008; De Jonghe et al., 2008; Ledenbach and Marshall, 2009; Ostrov et al., 2015). It was previously shown that the majority of isolates from raw milk from organic and conventional dairy farms belonged to the genus *Bacillus* and showed at least 97% 16S rRNA gene sequence similarity with type strains of *B. licheniformis*, *B. pumilus*, *B. circulans*, *B. subtilis* and *B. cereus* (Coorevits et al., 2008). Moreover, *B. cereus* and *B. licheniformis* were shown to be predominant species originated from dairy processing environments, raw materials, and processed foods, while *B. subtilis* was among prevalent heat-resistant and highly thermoresistant spore-formers (According to Lücking et al., 2013). *B. licheniformis* was shown to affect the quality of pasteurized milk and cream (Gilmour and Rowe, 1990). *B. cereus* was found to be responsible for sweet curdling (without pH reduction) both in homogenized and non-homogenized low-pasteurized milk (Andersson et al., 1995). *B. subtilis* has been associated with ropiness in raw and pasteurized milk as well as the spoilage of UHT and canned milk products (Heyndrickx and Scheldeman, 2002). Strains belonging to the *B. subtilis and B. cereus* groups were shown to be strongly proteolytic (Lücking et al., 2013). Interestingly, *Bacillus* strains including *B. cereus* (Wijman et al., 2007), *B. licheniformis* (Hoong et al., 2012; Ostrov et al., 2015) and *B. subtilis* (Bridier et al., 2011) are able to form submerged surface-associated biofilm.

Biofilm formation depends on the synthesis of an extracellular matrix that holds the constituent cells together. In *B. subtilis*, the model organism within the *Bacillu*s genus, the matrix has two main components, an exopolysaccharide (EPS) synthesized by the products of the *epsA-O* operon, and amyloid fibers encoded by *tasA* located in the *tapA-sipW-tasA* operon (Kearns et al., 2005; Branda et al., 2006; Chu et al., 2006; Vlamakis et al., 2013).

Since biofilm forming microorganisms in the dairy associated environment may hold spoilage and/or health risks, dairy products manufacturing is a subject to extremely stringent regulations (Bremer et al., 2006). The effective cleaning and disinfecting procedures in the dairy industry are a fundamental requirement to ensure the safety and quality of dairy products. Cleaning and disinfection in food manufacturing industries have been incorporated into the cleaning-in-place (CIP) regimes which include regular cleaning of processing equipment, usually with alkaline and acidic liquids at high temperatures (Zottola and Sasahara, 1994; Bremer et al., 2006). However, bacterial contamination and product spoilage due to biofilm formation are recurring problems (Carpentier et al., 1998). The result of the cleaning limitation of CIP procedures is accumulation of microorganisms on the equipment surfaces and formation of biofilm that is very difficult to remove by subsequent cleaning and disinfecting cycles (Peng et al., 2002; Hall-Stoodley et al., 2004). The biofilm formed by thermoresistant bacteria in a milk line can rapidly grow to such an extent that the passing milk is contaminated with cells released from the biofilm (Wirtanen et al., 1996).

Elimination of biofilm and spores is facilitated by a high degree of turbulence of the cleaning solution (Wirtanen et al., 1996) and by the presence of oxidizing substances such as hypochlorite and hydrogen peroxide (Kumar and Anand, 1998). Chlorine-based detergents can therefore facilitate the removal of biofilm. However, rapid recovery of biofilms after chlorine treatment is often observed. This may be due to the rapid regrowth of surviving cells, residual biofilm providing a conditioning layer for enhanced biofilm development, or selection of resistant microorganisms that survive and thrive after chlorine treatment (Flint et al., 1997). Currently, environmental concerns are driving dairy farms toward the use of chlorine-free detergents, although there is uncertainty about their effectiveness and thus it is difficult to judge whether this change can result in increased milk hygiene problems (Sundberg et al., 2011).

In order to ensure the optimal level of equipment hygiene in the dairy industry it is necessary to determine the removal efficiency of surface attached bacteria by cleaning solutions used for CIP procedures (Parkar et al., 2003; Bremer et al., 2006). However, currently there is no agreed standard method available for evaluating and comparing cleaning agents for use in CIP-procedures in the dairy industry under realistic conditions. Some progress has been made in a study which investigated the cleaning effects of various detergents under controlled realistic temperature and flow conditions (Sundberg et al., 2011). However, this study did not fully simulate the type of hygiene problems common in practice for instance the presence of extracellular matrix. Furthermore, previous studies could not evaluate the cleaning and disinfecting effect of the cleaning agents (whether the elimination effect is due to killing bacteria or removing them from the surfaces of dairy equipment). To this extent the necessity of not only killing bacteria in biofilms, but also removing them together with the extracellular matrix was previously emphasized (Zottola and Sasahara, 1994; Kumar and Anand, 1998; Parkar et al., 2003).

In the present study, we aimed to establish a model system to evaluate the effectiveness of cleaning agents in removal of biofilm derived spores from the surfaces of stainless steel which is predominant surface in milking equipment on dairy farms. Therefore, we developed a system in order to evaluate the cleaning outcome based on *Bacillus subtilis* spores surrounded with exopolymeric substances produced by bacteria during biofilm formation. The developed model system simulates actual farm conditions for quantitative evaluation of effectiveness of cleaning and disinfection agents and their cleaning and disinfecting effect on biofilm derived spores.

## Materials and Methods

### Strains and Growth Media

The *B. subtilis* wild type strain NCIB3610 (Branda et al., 2001) and its derivatives: RL3852 (Δ*epsH::*tet), YC668 (Δ*abrB*::kan) and YC189 (P*_tapA_ -cfp* at the *amy*E locus) (**Table 1**) were used in this study. The wild type strain was used for evaluation of effectiveness in removal of biofilm derived spores in the CIP model system as well as evaluation of cleaning/disinfecting effect of the cleaning agents and determining the role of the extracellular matrix in persistence of biofilm derived spores toward cleaning procedures. The RL3852 and YC668 strains were used for determining the role of the extracellular matrix in persistence of biofilm derived spores toward cleaning procedures. The YC189 was used for analysis of the level of the matrix gene expression. For routine growth, the strains were propagated in Lysogeny broth (LB; 10 g of tryptone, 5 g of yeast extract and 5 g of NaCl per liter) or on solid LB medium supplemented with 1.5% agar.

**Table 1:** Strains used in this study.

Strain	Genotype	Reference
*B. subtilis* NCIB3610	undomesticated WT strain	Branda et al., 2001
*B. subtilis* YC189	P*_tapA_-cfp* at the *amy*E locus in 3610, Spec^R^	Chai et al., 2008
*B. subtilis* RL3852	*ΔepsH* in 3610, Tet^R^	Kearns et al., 2005
*B. subtilis* YC668	*ΔabrB* in 3610, Kan^R^	Pasvolsky et al., 2014


### Generation of Biofilm Derived Spores

**FIGURE 1:**
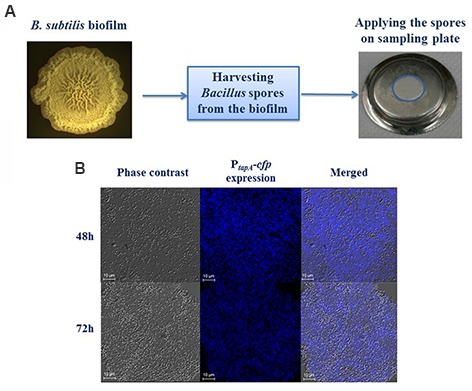
**Generation of *Bacillus subtilis* biofilm derived spores. (A)** Schematics showing that the spores, harvested from biofilm of *B. subtilis* 3610 (wild type strain) generated on LBGM agar plates, were mounted on the sampling plate. **(B)** Transcription of the *tapA-sipW-tasA* operon responsible for the matrix production in *B. subtilis* is greatly upregulated in LBGM following either 48 or 72 h of biofilm development. Intensity of the *tapA* expression by the cells that bear the P*_tapA_* -*cfp* transcriptional fusion (*B. subtilis* YC189) is presented in the middle-hand panel. The biofilm forming cells were obtained as previously reported (Shemesh and Chai, 2013) and analyzed using confocal laser scanning microscope (CSLM, Leica, Germany) visualization. Scale –10 μm.

Biofilm colonies were generated at 30°C in biofilm promoting medium LBGM [LB + 1% (v/v) glycerol + 0.1 mM MnSO_4_] (Shemesh and Chai, 2013) (**Figure 1A**). The grown colonies were collected and suspended in phosphate buffer saline (PBS; 0.01 M phosphate buffer, 0.0027 M KCl, 0.137 M NaCl per 200 ml, Sigma Aldrich, St. Louis, MO, USA). Chained and bundled cells from the collected biofilm colony were disrupted by mild sonication (amplitude–50%, pulse–10 s, pause–5 s, duration–1.5 min.). Then, heat killing was performed at 80°C for 20 min. Cell numbers after heat killing were quantified by the plating method using LB agar plates.

### Preparation and Enumeration of the Spores on the Sampling Plates

Prior to the cleaning tests, 200 μl portions from the suspension of spores (prepared as described above) were applied on each sampling plate and carefully distributed over the sampling area (**Figure 1A**). The goal was to attach approximately two million spores onto each plate. The plates were then placed upright in biological laminar hood to dry for around 1 h. Two or three sampling plates were prepared but not cleaned in the test installation. Otherwise, the subsequent treatment of these control plates was precisely the same as for the cleaned plates. Average spore counts on the control plates were used as the initial value for all plates cleaned on that day when calculating the level of spore reduction.

For enumeration of spores, the sampling area on each plate was carefully swabbed with cotton swabs moistened in PBS buffer. Swabs from each plate were then agitated in PBS in separate test tubes. Serial dilutions from each sample were prepared, followed by spread plating on LB agar for CFU analysis. Plates were incubated for 24 h at 37°C before colonies were counted.

### Cleaning Solutions

We choose to use for this study caustic soda (NaOH, pH value–13) and five different commercial alkaline detergents (defined as solutions A–E; pH value between 11–12) which are commonly used in the Israeli dairy farms. All detergents were used at concentration of 0.5% (v/v) in accordance with the manufacturer’s recommendations. Caustic soda was used at concentration of 0.5% (m/v). As a control, tap water was used (pH value around 7.7) with a standard level of hardness (~50 mg/l Ca^2+^, 50 mg/l Mg^2+^) without addition of any detergent.

### Test Installations

**FIGURE 2:**
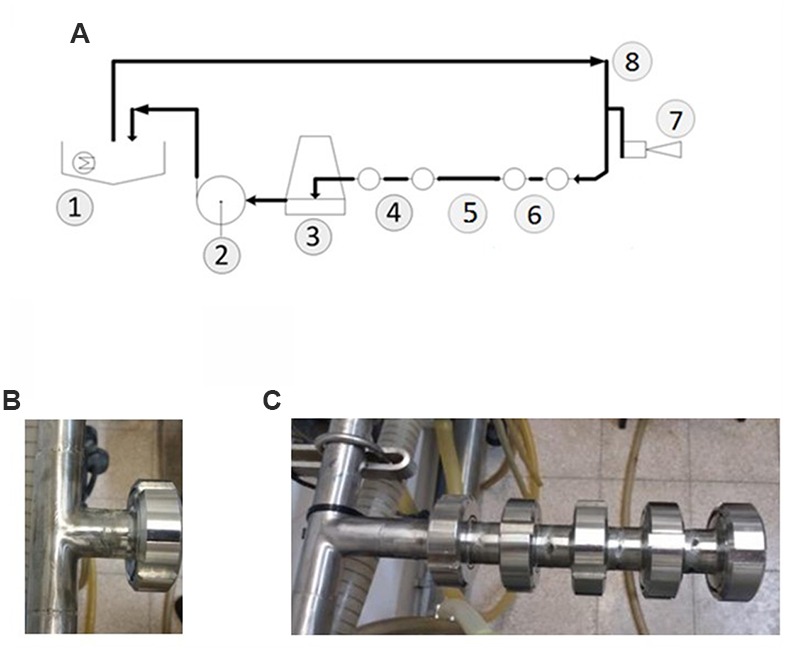
**Description of the developed CIP-model system. (A)** Schematic design of the model system used in the cleaning tests: (1) sink with the heater, (2) milk pump, (3) releaser, (4) two 35 mm T-junctions, (5) the mane pipeline, Ø25 mm, (6) two 75 mm-T junctions, (7) air injection, (8) pipeline of water supply from the sink and the flow direction of circulating liquid. **(B)** The shortest T-junction (35 mm). **(C)** 75 mm T-junction elongated using removable stainless steel nozzles to the total length of 275 mm.

The cleaning tests were carried out in the CIP-model system which was designed to resemble farm conditions as closely as possible. The main components were a 5-m stainless steel milk line (25 mm internal diameter; fitted with a test outfit) for pumping the cleaning agents from the basin (**Figure 2A**), milk releaser, and stainless steel return line to the basin. To generate flushing pulsation of the circulating liquid, air was introduced into the milk line at controlled intervals (every 8 s, Supplementary Video 1). The test outfit had removable sampling plates, attached at the end of T-junctions that protruded either 75 mm or 35 mm from the main loop. In order to reflect different degrees of cleaning difficulty (characteristic for dairy equipment), removable stainless steel nozzles were used to increase the length of T-junctions in certain cleaning tests. The length of each nozzle constituted 50 mm. Thereby, the length of each T-junction could be increased by 50–200 mm (**Figures 2B,C**). The sampling plates were made from stainless steel (304) and the sampling area exposed to the cleaning solutions was about 5.7 cm^2^. The temperature of the cleaning agent during the cleaning tests constituted 50°C. The flow rate with air injection was 34.5 l per minute; the flow rate without air injection was 43 liter per minute. The duration of each cleaning cycle was 10 min.

### Evaluation of the Effect of the Cleaning Agents on the Viability of *B. subtilis* Spores

The tested detergents (0.5%, v/v) and caustic soda (0.5%, m/v) were added to spore suspension of *B. subtilis* 3610 containing around 1 × 10^7^ CFU/ml spores. Whereas, the spore suspension within water without addition of detergents was used as control. The samples were incubated in closed tubes at conditions simulating those in CIP-model system (50°C, 200 rpm) for 30 min. The CFU-measurements of the number of viable spores were made every 10 min.

### Statistical Analysis

Student’s *t* test was used to calculate the significance of the difference between the mean expression of a given experimental samples and the control samples. A *P* value of < 0.05 was considered significant.

## Results

### Developing a Model Based on Biofilm Derived Spores

In order to simulate biofilm derived spores, we have developed the system that is based on *B. subtilis* spores surrounded with exopolymeric substances produced by bacteria during biofilm formation. To stimulate the sporulation in biofilm context, we generated *B. subtilis* colonies in the biofilm promoting medium LBGM (**Figure 1A**). To confirm the high level production of extracellular matrix in the biofilm colonies, we analyzed the level of the matrix gene expression in LBGM using transcriptional fusion of the promoter for *tapA-sipW-tasA* to the *cfp* gene encoding cyan fluorescent protein (Chai et al., 2008) similarly as described previously (Shemesh et al., 2010). We found that the expression of the P*_tapA_-cfp* was enhanced in a large number of cells both after 48 and 72 h of biofilm development (**Figure 1B**). This finding indicates that *B. subtilis* spores harvested from biofilm colonies could be surrounded with extracellular polymeric substances.

### Evaluation of Effectiveness of Biofilm Derived Spores Removal in the CIP Model System

It was suggested that the hydrodynamic effects such as turbulent flow of cleaning agent may facilitate the removal of surface associated bacteria in dairy equipment (Wirtanen et al., 1996; Leliévre et al., 2002, 2003). However, dairy equipment has many so called “dead legs” (milk meters, clusters, etc.) protruding from the main pipelines in which the flow of liquid is much less turbulent. Such “dead legs” might represent higher levels of cleaning difficulty compared to other sites of dairy equipment. To simulate different levels of cleaning difficulty characteristic for dairy equipment in the CIP model system, we used removable stainless steel nozzles to increase the length of T-junctions (**Figures 2B,C**). We hypothesized that the level of efficiency of cleaning agents toward removal of biofilm derived spores is inversely proportional to the length of T-junctions (Supplementary Video 2).

**FIGURE 3:**
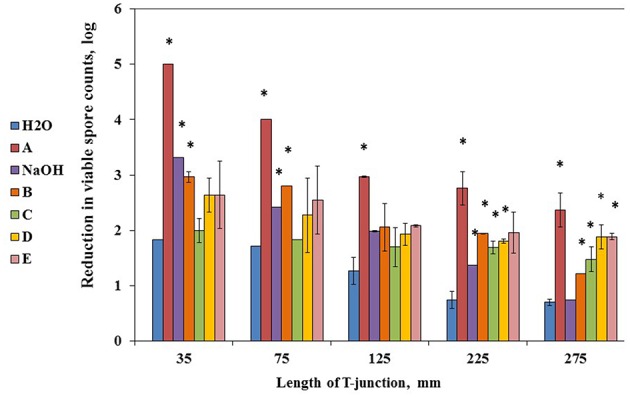
**Mechanical effect of water and chemical/biological effect of cleaning agents on removal of biofilm derived spores in the CIP-model system.** Sampling plates, each maintaining approximately 2 million spores of *B. subtilis* wild type strain, were mounted on T-junctions protruding 35, 75, 125, 225, and 275 mm from the main loop of the CIP model system, and cleaned in the installation. Caustic soda (NaOH) and five different commercial alkaline detergents (defined as A–E) were used as the cleaning agents. The detergents were dosed as 0.5% (v/v) in accordance with the manufacturer’s recommendations. Caustic soda was dosed as 0.5% (m/v). As a control, tap water without addition of any detergent was used. The cleaning effect was evaluated by comparing the numbers of viable spores (attached to sampling plates) before and after cleaning. The results represent the means and standard deviation (SD) of two independent biological experiments performed in duplicates.^∗^statistically significant difference (*P* < 0.05) between reduction in viable spore counts in given sample versus reduction in spore counts after cleaning with water (control).

Primarily, we evaluated mechanical effect of water circulation in the CIP system. Cleaning with water alone reflects the mechanical cleaning effect brought about by the flow of liquid in the installation (Sundberg et al., 2011). The difference in cleaning effect between water and a cleaning agent reflects the chemical/biological effect from the substances present in the agent. We found that effectiveness of water in removal of biofilm derived spores was inversely proportional to the length of T-junctions and constituted about 1.8 and 1.7 log reduction in spore counts for 35- and 75-mm T-junctions, respectively; while 1.3 log reduction for 125-mm T-junctions and about 0.7 log reduction for 225- and 275-mm T-junctions, respectively (**Figure 3**). These results confirm that mechanical effect of flow turbulence facilitates the removal of biofilm derived spores.

Next, spores removal efficiency of caustic soda and five different commercial alkaline detergents with chlorine was determined. It was shown that chemical/biological effect of the tested detergents constituted additional 0.5–2 log reduction compared to mechanical effect of water circulation. Among all tested detergents, solution A had the highest removal efficiency leading to additional 2 log reduction in spore counts irrespective of the length of T-junctions (**Figure 3**).

As the water circulates by flushing pulsation in the commercial cleaning units, we tested the effect of air introduction into the CIP system on the spores removal efficiency of the tested agents. Our experiments established that there was no significant difference (*P* < 0.26) in the removal efficiency without the air introduction into the milk line (data not shown). We also tested the effect of temperature on the removal efficiency. We found about 0.5 log improvement in the efficiency of cleaning out biofilm derived spores by elevating the temperature from 35°C to 50°C (data not shown).

### Evaluation of the Cleaning and Disinfecting Effect of the Cleaning Agents

**FIGURE 4:**
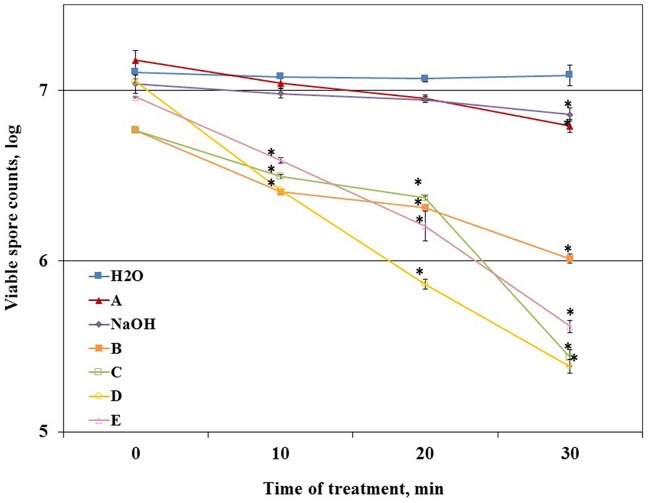
**The effect of the tested cleaning agents on the viability of *B. subtilis* spores.** Caustic soda (NaOH) and five different commercial alkaline detergents (defined as A–E), were added to the tubes with spore suspension of WT *B. subtilis* within distilled sterile water, containing approximately 10^7^ CFU/ml spores. The detergents were dosed as 0.5% (v/v) in accordance with the manufacturer’s recommendations. Caustic soda was dosed as 0.5% (m/v). Spore suspension without any detergent was used as control. The samples were incubated at 50°C for 30 min. The ability of detergents to eradicate spores (disinfecting effect) was evaluated by comparing the numbers of viable spores in control and after the treatment with a tested detergent at different time points of incubation. The results represent the means and standard deviation (SD) of two independent biological experiments performed in duplicates.^∗^statistically significant difference (*P* < 0.05) between viable spore counts in given sample versus spore counts after cleaning with water (control).

Primarily, we determined the ability of the tested agents to reduce the number of viable spores (disinfecting effect). For this, *B. subtilis* spores suspensions were incubated with each of the tested agents in conditions simulating those in the CIP-model system. We found that solution A and caustic soda could not notably reduce the spore counts compared to control (about 0.2 log after 30 min of incubation) (**Figure 4**). At the same time, other tested agents leaded to noticeable reduction (about 0.5 log) in the number of viable spores even after 10 min of incubation.

**FIGURE 5:**
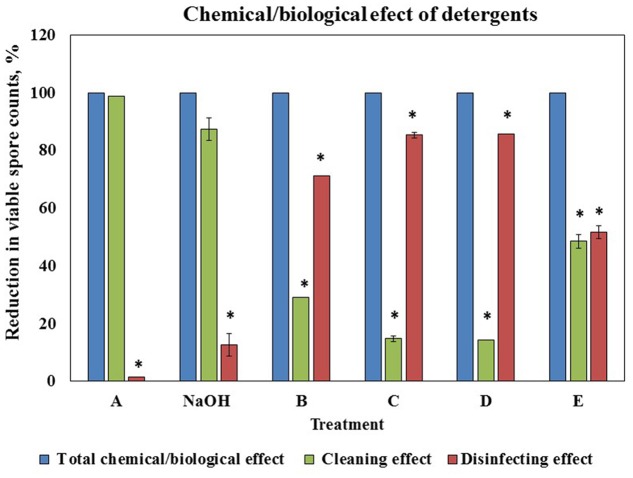
**Correlation between cleaning and disinfecting effect of the tested agents.** The ability of a cleaning agent to reduce the number of viable spores (*B. subtilis* wild type strain) after 10 min of incubation (as the duration of cleaning cycle in the CIP model system is 10 min) was defined as disinfecting effect. The percentage of the disinfecting effect was compared to the total chemical/biological effect of a cleaning agent determined in the CIP system after 10 min of cleaning test conduction (taken as 100%). The difference between the total chemical/biological effect of a cleaning agent and disinfecting effect was defined as cleaning effect. The results represent the means and standard deviation (SD) of two independent biological experiments performed in duplicates. ^∗^statistically significant difference (*P* < 0.05) between reduction in spore counts due to cleaning or disinfecting effect versus total chemical/biological effect of a tested agent.

To determine a correlation between the cleaning and disinfecting effect of the tested detergents we defined the ability of a cleaning agent to reduce the number of viable spores after 10 min of incubation (as the duration of cleaning cycle in the CIP model system is 10 min) as disinfecting effect. We compared the percentage of the disinfecting effect to the total chemical/biological effect of a cleaning agent determined in the CIP system after 10 min of cleaning test conduction (taken as 100%). The difference between the total chemical/biological effect of a cleaning agent and disinfecting effect was defined as cleaning effect. It was established that chemical/biological effect of solution A and caustic soda was mostly due to removal of surface attached spores, solution E was characterized by approximately equal cleaning and disinfecting properties; while chemical/biological effect of B, C, and D was mostly due to disinfecting (**Figure 5**).

### Determining the Role of the Extracellular Matrix in Persistence of Biofilm Derived Spores toward Cleaning Procedures

**FIGURE 6:**
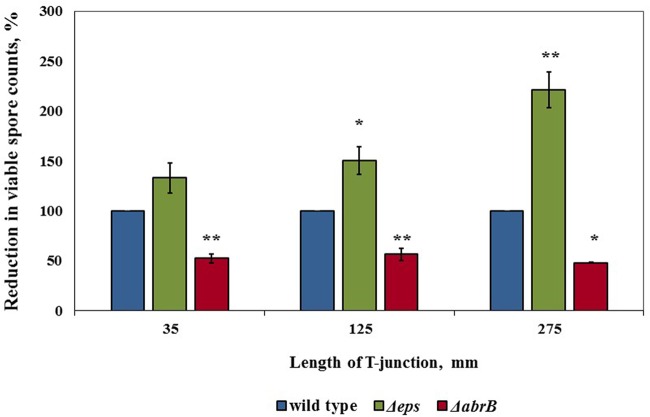
**The role of extracellular matrix in persistence of biofilm derived spores against removing by cleaning procedures.** Sampling plates, each maintaining approximately 2 million spores of WT *B. subtilis*, *B. subtilis* RL3582 (*ΔepsH*), *B. subtilis* YC668 (*ΔabrB*), were mounted on T-junctions protruding 35, 125, and 275 mm from the main loop of the CIP model system, and cleaned in the installation. Tap water without addition of any detergent was used as a cleaning agent. The cleaning effect was evaluated by comparing the numbers of viable spores (attached to sampling plates) before and after cleaning. The cleaning effect for *B. subtilis* 3610 was taken as 100%. The results represent the means and standard deviation (SD) of two independent biological experiments performed in triplicates.^∗^statistically significant difference (*P* < 0.05); ^∗∗^statistically significant difference (*P* < 0.006) between reduction in viable spore counts in given sample versus reduction in spore counts in control wild type bacteria.

To support the assumption that there is an extracellular matrix around the spores which may provide a protection, we evaluated mechanical effect of water circulation toward the spores produced by *ΔepsH* strain of *B. subtilis* (this mutant strain cannot produce exopolysaccharide component of extracellular matrix). As we hypothesized, there was a notable increase in reduction of viable spore counts for Δ*epsH* compared to wild type strain for the two higher lengths of the T-junctions (**Figure 6**). Furthermore, we evaluated the mechanical effect of water circulation toward the spores produced by the Δ*abrB* of *B. subtilis* (this mutant strain overproduces extracellular matrix). We found that it was far more difficult to remove the spores of Δ*abrB* strain compared to wild type (**Figure 6**). These results indicate that the presence of extracellular matrix is an important factor responsible for high levels of cleaning difficulty.

## Discussion

It becomes increasingly clear that the major source of the contamination of dairy products is often associated with attached bacteria on the surfaces of dairy processing equipment (Flint et al., 1997). Thus, there is a need to develop a model system for evaluating and comparing the effectiveness of cleaning agents in removal of attached bacteria from the surfaces of stainless steel under realistic conditions.

This study, investigated the removal efficiency of caustic soda and commercial alkaline detergents toward biofilm derived spores using a developed CIP model system under well-controlled realistic temperature and flow conditions.

We used *Bacillus* spores as a model, because of their high adherence to various materials (Faille et al., 2001) and their resistance to heat and chemicals (Faille et al., 2002). Several previous studies investigated cleaning efficiency during CIP procedures (Leliévre et al., 2003; Sundberg et al., 2011; Faille et al., 2013) using *Bacillus* spores as a model. However, previous models do not fully reflect the type of hygiene problems common in practice such as the presence of extracellular matrix of biofilm origin. The conditions, encountered in the dairy equipment are often propitious for bacterial growth and eventually a biofilm is formed. Previous works have demonstrated that sporulation could occur in biofilms, suggesting that biofilms would be a significant source of food contamination with spores (Wijman et al., 2007; Faille et al., 2014). Consequently, the spores derived from biofilm represent continuous microbial problem which could be very hard to eliminate partially due to the presence of extracellular matrix that might influence their resistance during cleaning procedures.

Our results show that spores removing efficiency during cleaning procedures was inversely proportional to the length of T-junctions (**Figure 3**). This is in consistence with previous papers suggesting that turbulence may influence removal of surface attached bacteria (Wirtanen et al., 1996; Leliévre et al., 2002, 2003). It is generally considered that a “dead leg” is cleanable when the flow is directed into the “dead leg” and its length does not exceed twice the diameter of the pipeline (Chisti, 1999). In our study, the T-junctions were 35–275 mm that is 1.5–11-times the diameter of the pipeline. Therefore, it is conceivable that we observed notable decrease in effectiveness of spores elimination by the tested agents with the increase of the length of T-junctions. These results confirm that the developed CIP model system simulates different levels of the cleaning difficulty that facilitates proper evaluation of spores elimination effectiveness of cleaning agents at realistic conditions.

Interestingly, our experiments demonstrated that there was no significant difference in spores removal efficiency without the air introduction into the milk line. This could be explained by the relatively low diameter of pipeline which was used in the developed system. Most likely, the differences in flow rate and turbulence were not significant with or without introduction of air.

Moreover, we found around 0.5 log improvement in the spores removal efficiency by elevating the temperature from 35–50°C. This finding is also in consistence with previous studies which demonstrated dependence of the cleaning efficiency on temperature (Peng et al., 2002; Sundberg et al., 2011). Taken together, our results suggest that elevated temperature as well as chemical/biological effect may help to eliminate biofilm derived spores in milking equipment.

The methods of evaluation of cleaning effectiveness described earlier (Parkar et al., 2003; Bremer et al., 2006; Sundberg et al., 2011) do not show if chemical/biological effect of cleaning agents is due to killing bacteria (disinfecting effect) or to removing them from the surfaces of dairy associated equipment (cleaning effect). The necessity of not only killing bacteria in biofilms, but also removing the immobilized bacteria is suggested (Flint et al., 1997; Kumar and Anand, 1998; Parkar et al., 2003) as rapid recovery of biofilms after disinfectant treatment is often observed. Therefore, we developed a method to evaluate the cleaning and disinfecting effect of cleaning agents toward biofilm derived spores. Using this approach it is shown whether chemical/biological effect of a detergent is due to cleaning, disinfecting or both.

In conclusion, a CIP model system was developed and used to evaluate the efficiency of cleaning agents in removing biofilm derived spores from the surfaces of dairy equipment. The developed system simulates actual farm conditions for proper evaluation of the spores elimination effectiveness and cleaning and disinfecting effect of cleaning and disinfection agents.

## Author Contributions

IO and MS planned the experiments and wrote the original manuscript. IO performed the experiments described in the manuscript. AH and MS designed the CIP-model system described in the manuscript. AH and SB provided technical assistance for conduction of experiments. DS revised the manuscript. IO, DS, and MS integrated all of the data throughout the study and crafted the final manuscript.

## Conflict of Interest Statement

The authors declare that the research was conducted in the absence of any commercial or financial relationships that could be construed as a potential conflict of interest.
